# Emergence of *Mycobacterium simiae*: A retrospective study from a tertiary care center in Lebanon

**DOI:** 10.1371/journal.pone.0195390

**Published:** 2018-04-04

**Authors:** Amal Hamieh, Ralph Tayyar, Houssam Tabaja, Saeed E. L. Zein, Pierre Bou Khalil, Nathalie Kara, Zeina A. Kanafani, Nadim Kanj, Imad Bou Akl, George Araj, Ghina Berjaoui, Souha S. Kanj

**Affiliations:** 1 Department of Internal Medicine, Division of Infectious Diseases, American University of Beirut Medical Center, Beirut, Lebanon; 2 Department of Internal Medicine, Division of Pulmonary and Critical Care Medicine, American University of Beirut Medical Center, Beirut, Lebanon; 3 Department of Pathology and Laboratory Medicine, American University of Beirut Medical Center, Beirut, Lebanon; 4 Department of Radiology, American University of Beirut Medical Center, Beirut, Lebanon; Hospital Universitari de Bellvitge, SPAIN

## Abstract

**Objective:**

The objective of this study is to describe the clinical significance of *Mycobacterium simiae* at a major tertiary care center in Lebanon.

**Methods:**

This is a retrospective study of patients with positive cultures for *M*. *simiae* isolated between 2004 and 2016 at the American University of Beirut Medical Center.

**Results:**

This study included 103 *M*. *simiae* isolates recovered from 51 patients. Their mean age was 62.7 years. The majority were males and smokers. Specimens were mostly from respiratory sources (97%). Common comorbidities included chronic lung disease (such as chronic obstructive pulmonary disease), solid tumor, systemic disease, and diabetes mellitus. Productive cough and dyspnea were the most common symptoms. Frequent radiographic findings were infiltrates and nodules on chest X-ray and nodules, infiltrates, and bronchiectasis on chest computed tomography scan. Among 18 tested isolates, 5.8% were resistant to clarithromycin, 11.7% to amikacin, and 70–100% to other antimicrobials. Out of 13 patients receiving early treatment, 5 noted improvement, one had recurrence of symptoms, two received alternative diagnosis, and five died. Two of those deaths were related to *M*. *simiae*. Common treatment regimens included clarithromycin in different combinations with trimethoprim-sulfamethoxazole, moxifloxacin, and amikacin. Moreover, clofazimine was used in only two patients whose isolates were resistant to all but one agent. Duration of treatment ranged from 6–24 months.

**Conclusion:**

In Lebanon, *M*. *simiae* is increasingly encountered with true infection rates of at least 47%. Furthermore, the prevalence of multidrug resistance among the Lebanese *M*. *simiae* isolates is very high limiting the treatment options.

## Introduction

Non-tuberculous mycobacteria (NTM), or environmental mycobacteria, were first discovered from clinical samples in 1885 [1]. This family differs from *Mycobacterium tuberculosis* complex and *M*. *leprae* in its clinical spectrum, diagnostic criteria, and treatment modalities. Currently, more than 160 species of NTM have been recognized. The majority are found arbitrarily in the environment and regarded as non-pathogenic [2]. *M*. *simiae*, one of the slowest growing NTM, was previously described in the Southern United States, Cuba, Palestine, Iran, Israel, Turkey, and Japan [3]. It was initially identified from rhesus monkeys in 1965 [4]. Data from the microbiology laboratory at the American University of Beirut Medical Center (AUBMC), suggests that among speciated isolates, *M*. *simiae* is the most frequently isolated NTM over the past 2 decades, ranging from 37% to 65% (published in the yearly AUBMC bacteriology report). As in other studies most isolates were from respiratory specimens [5].

The *M*. *simiae* complex group includes 18 species: *M*. *intermedium*, *M*. *interjectum*, *M*. *kubicae*, *M*. *montefiorense*, *M*. *florentinum*, *M*. *sherrisii*, *M*. *parmense*, *M*. *parascrofulaceum*, *M*. *lentiflavum*, *M*. *triplex*, *M*. *heidelbersengh*, *M*. *palustre*, *M*. *genavense*, *M*. *simiae*, *M*. *saskatchewanense*, *M*. *stomatepiae*, *M*. *eurapaeum*, *and M*. *paraense*. [6, 7, 8, 9]. *M*. *simiae* is the most common species in the complex to cause human pathology [10]. It has been identified from soil and water sources [4, 11], such as local tap water [2]. To date, there are no proven cases of human-to-human or animal-to-human transmission [2].

NTM are abundant in the environment and are potential contaminants of medical instruments and laboratory isolates. Therefore, their isolation from clinical samples does not always represent a true infection [12]. In 2007, the American Thoracic Society (ATS) issued guidelines to differentiate between true and pseudo-NTM infections [4]. It was estimated that only 9 to 21% of isolated *M*. *simiae* are clinically relevant [4]. In Lebanon, the epidemiology and clinical impact of *M*. *simiae* have not yet been described. The aim of this study is to shed light on *M*. *simiae* infections at one of the major tertiary care centers in Lebanon, with regards to epidemiology, clinical manifestations, radiographic findings, susceptibility profile of isolated pathogens, treatment approach, and response to therapy.

## Materials and methods

This is a descriptive retrospective study that included patients with positive cultures for *M*. *simiae* isolated from respiratory and non-respiratory specimens between January 2004 and April 2016 at AUBMC. This study was approved by the Institutional Review Board (IRB) at AUBMC. In accordance with the IRB regulations, verbal informed consent was obtained from all patients or their legal representative before medical record review.

### Patients

All patients with positive *M*. *simiae* cultures were eligible to be recruited in the study. This study focused on adults because no pediatric *M*. *simiae* isolates were encountered. *M*. *simiae* was isolated from respiratory specimens (sputum, bronchoalveolar lavage [BAL], lung biopsy, pleural fluid), or non-respiratory specimens (cerebrospinal fluid). Isolates from a respiratory specimen had to meet the ATS criteria to be considered as the causative pathogen for the pulmonary disease. Demographic, clinical, and microbiological data were retrieved from the patients’ medical records. Missing data were either provided by the treating physicians or the patients. A phone call follow-up was performed at the end of the data collection phase to gather information about any change in symptoms, initiation, and total duration of antibiotic intake for those who were treated.

All relevant chest X-rays and chest computerized tomography (CT) scans were reviewed by a radiologist at AUBMC.

### Specimens

All specimens were submitted for mycobacterial cultures at the Clinical Microbiology Laboratory at AUBMC. They were processed according to standard procedures by inoculation on Lowenstein-Jensen media (Becton Dickinson Microbiology system) and Middlebrook 7H12 broth medium (BACTEC 12B/ MGIT Mycobacteria Growth Indicator Tube media) and incubated for 4–6 weeks [5]. The differentiation between NTM and *M*. *tuberculosis* was done using the BD Mycobacteria Growth Indicator Tube TBc (BDMGITTBc) identification test. NTM isolates were then referred to Mayo Clinic for speciation and drug susceptibility testing. Speciation relied on the MALDI-TOF technique and/or DNA sequencing performed in Bioscientia institut fur Medizinische diagnostic GmbH, Germany and Mayo Clinic laboratories–Rochester main campus, Minnessota, USA.

The susceptibility profile was determined at Mayo Clinic using a manual (microdilution) method for the following drugs: moxifloxacin, ciprofloxacin, clarithromycin, trimethoprim/sulfamethoxazole (TMP/SMX), streptomycin, amikacin, isoniazid, ethambutol, rifampin, rifabutin, and linezolid. Clofazimine susceptibility was performed on two specimens only, which were resistant to all but one drug.

### Statistical analysis

IBM SPSS Statistics version 23 was used to analyze the data in this series.

## Results

### Demographics

A total of 51 patients were included in this study. All but three patients were Lebanese, with a male predominance (55%). Most patients were from the capital Beirut (60.7%) followed by Mount Lebanon (11.7%). The mean age was 62.7 ± 15.8 years and a large proportion of patients were smokers (53%). *M*. *simiae* was isolated from a total of 103 specimens and the distribution was as follows: sputum (76/103; 74%), BAL (24/103; 23%), lung biopsy (1/103; 0.97%), pleural fluid (1/103; 0.97%), and CSF (1/103; 0.97%). The most frequent comorbidities were structural lung diseases including chronic obstructive pulmonary disease (COPD) (24%), asthma (8%), and interstitial lung disease (6%). Moreover, non-pulmonary comorbid diseases included solid tumors (12%), systemic diseases (10%), diabetes mellitus (11%), and heart failure (8%). Demographic data is provided in Table 1.

**Table 1:** Demographics and clinical manifestations of patients with positive cultures for *M*. *simiae*.

Patients characteristics	Patients with positive findings (%)*	Patients meeting diagnostic criteria among those with positive findings*
**Mean Age ± SD (years)**	62.7 ± 15.8	68 ± SD
**Male Gender**	28/51 (55)	12/28 (43)
**Smoking**	23/43 (53)	13/23(57)
**Non lung related comorbidities**		
Solid tumor	6/49 (12)	2/6 (33)
DM	5/46 (11)	5/5 (100)
Systemic disease (Ulcerative colitis, Rheumatoid arthritis, Sarcoidosis)	5/51 (10)	4/5 (80)
CHF	4/51 (8)	2/4 (50)
CKD	2/51 (4)	2/2 (100)
Stem cell transplant	1/51 (2)	1/1 (100)
**Lung related comorbidities**		
Recurrent pneumonia	15/49 (31)	10/15 (67)
COPD	12/51 (24)	8/12 (67)
Asthma	4/51 (8)	2/4 (50)
ILD	3/49 (6)	2/3 (67)
Latent TB	2/ 51 (4)	1/2 (50)
Pleural disease	1/51 (2)	1/1 (100)
**Clinical manifestations**		
Symptomatic	33/46 (72)	
Cough	33/33 (100)	20/33 (61)
Sputum production	30/33 (91)	19/30 (63)
Dyspnea	19/33 (58)	9/19 (47)
Hemoptysis	9/33 (27)	3/9 (33)
Fever	7/33 (21)	3/7 (43)
Weight loss	7/33 (21)	2/7 (29)
Night sweats	5/33 (15)	3/5 (60)

SD: standard deviation, CHF: congestive heart failure, DM: diabetes mellitus, CKD: chronic kidney disease, ILD: interstitial lung disease, COPD: chronic obstructive pulmonary disease.

*Numbers indicate n/N (%) unless otherwise specified. Patients meeting diagnostic criteria include those with positive *M*. *simiae* cultures who meet the ATS criteria for the diagnosis of infection.

### Clinical aspects

#### Clinical manifestations

Data on clinical symptoms was available for 90% (46/51) of patients, of which 72% were symptomatic. Among symptomatic patients, the most frequently reported symptoms were cough (100%), sputum production (91%) and dyspnea (58%) (Table 1). Notably, some symptomatic patients failed to meet ATS criteria for true NTM infection. Around 70% of the patients had clinical symptoms that were attributed to *M*. *simiae* infection. The patients’ clinical manifestations are listed in Table 1. Forty-nine patients (96.1%) had *M*. *simiae* isolated from the respiratory tract only (sputum, BAL, or pleural fluid). In 12 of the patients, *M*. *simiae* was recovered from both sputum and BAL cultures. One patient had *M*. *simiae* recovered from pleural fluid as well as from sputum. Only one patient (2%) had extra-pulmonary *M*. *simiae*, isolated from the CSF; this patient was also described by Balkis et al. in 2009 [6].

#### Radiology

**Fig 1:**
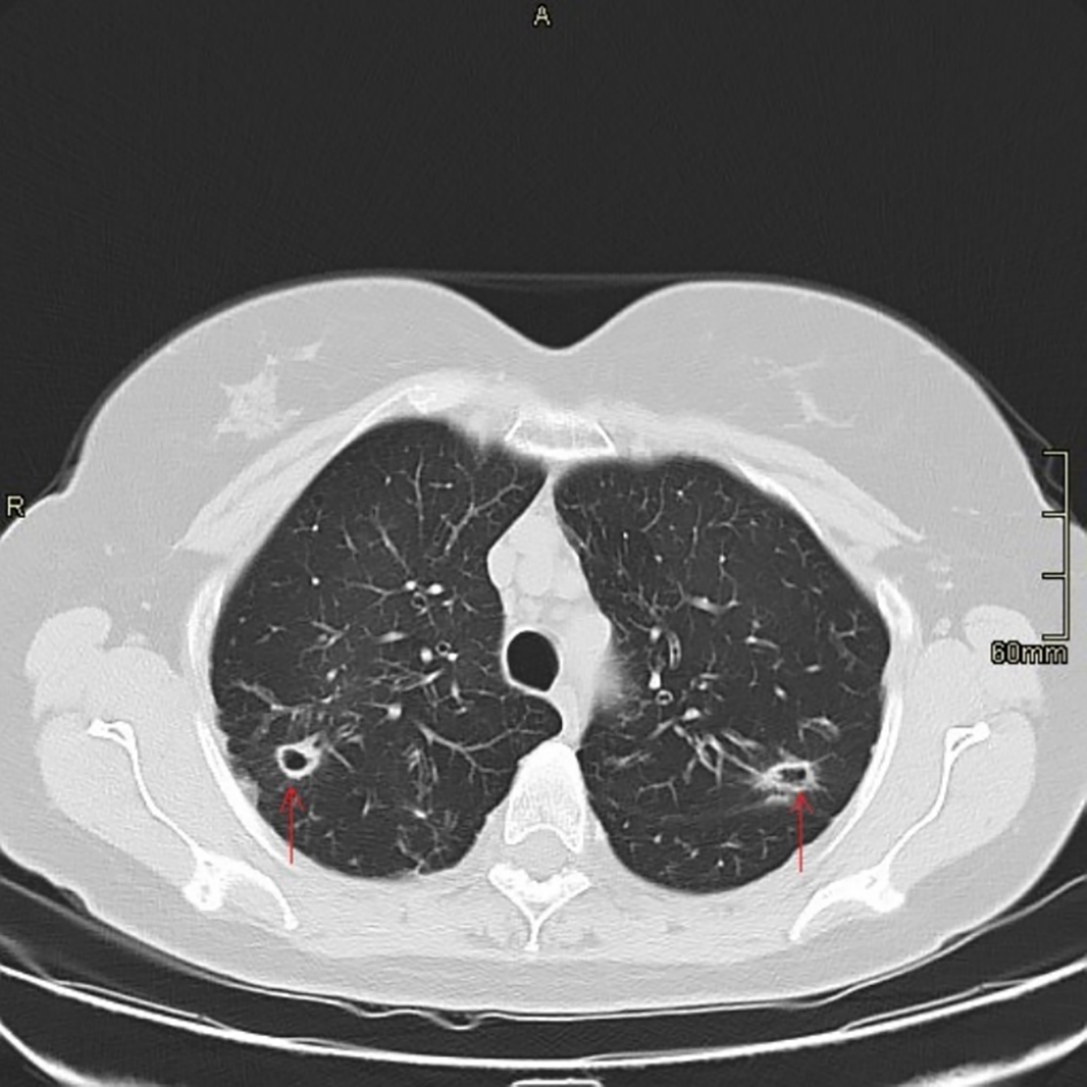
CT chest showing cavitary nodules.

**Fig 2:**
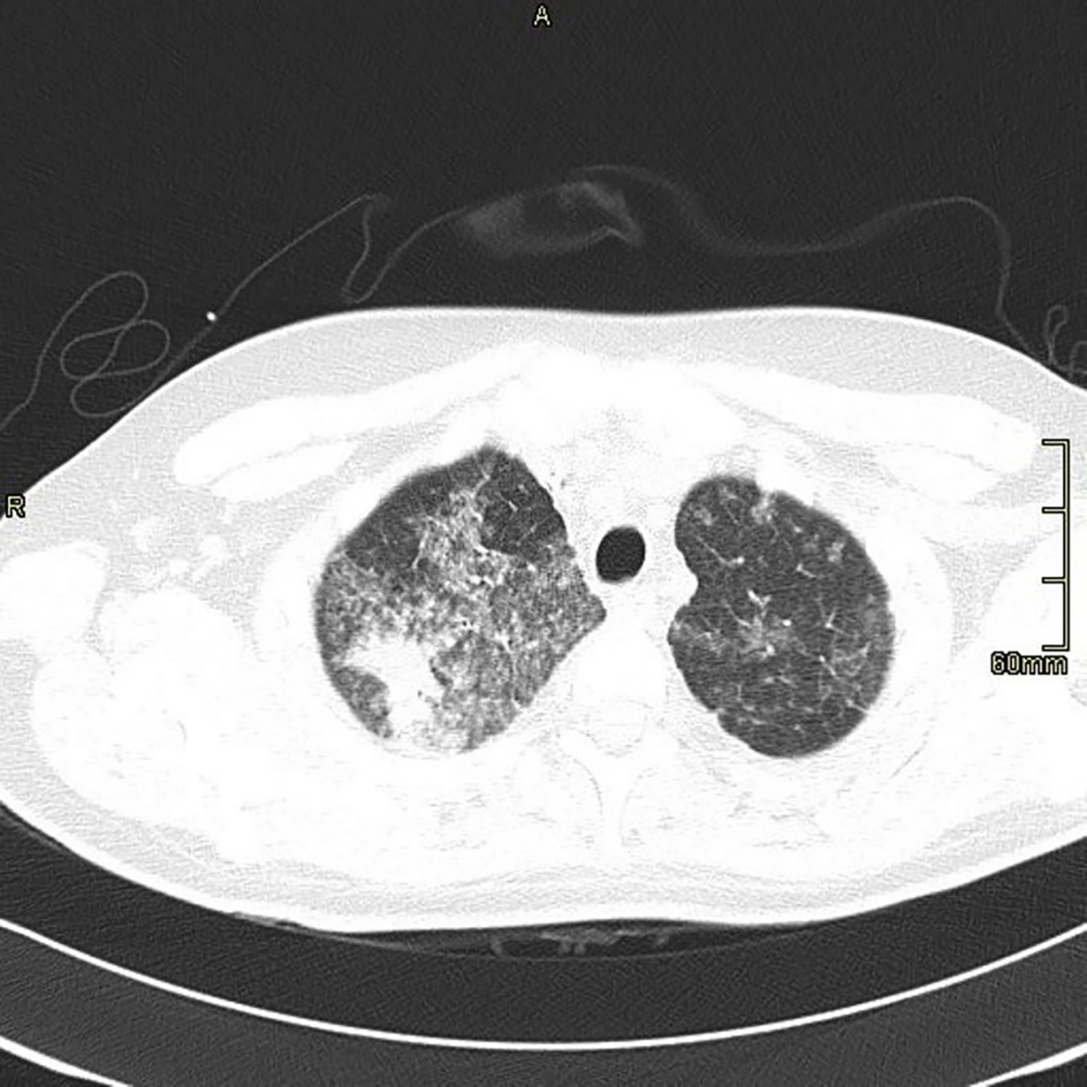
CT chest showing patchy ground glass opacities with right apical consolidation.

Table 2 is a summary of the radiological findings. Based on high resolution CT scans, the most common radiographic findings in the patient population included nodular lesions (61%) (Fig 1), infiltrates (50%), bronchiectasis (34%), consolidations (30%), and ground glass infiltrates (25%) (Fig 2), with almost equal involvement of the upper and lower lobes. Moreover, most of the radiological findings were seen in patients who met the ATS criteria.

**Fig 3:**
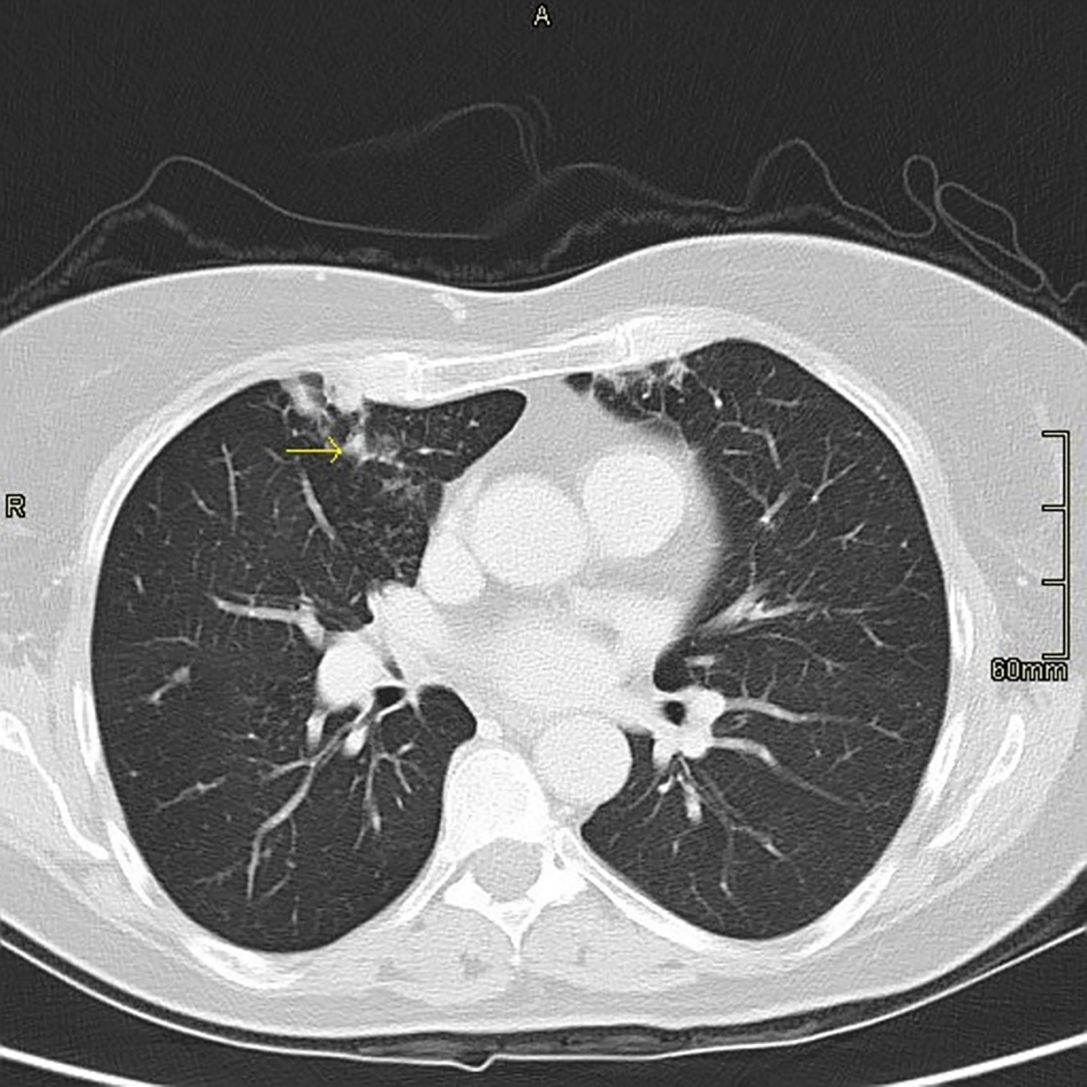
CT chest showing tree-in-bud infiltrates and diffuse nodularity.

**Table 2:** Radiographic findings in patients with positive cultures for *M*. *simiae*.

Radiology	Patients with positive findings (%)*	Patients meeting diagnostic criteria among those with positive findings*
**Chest X-Ray**		
Infiltrates	7/22 (32)	5/7 (71)
Nodules	5/22 (23)	2/5 (40)
Pleural effusion	2/22 (9)	2/2 (100)
Fibrotic changes	2/22 (9)	2/2 (100)
Consolidation	1/22 (5)	1/1 (100)
Cavitations	1/22 (5)	1/1 (100)
Pleural thickening	1/22 (5)	1/1 (100)
Emphysema	1/22 (5)	1/1 (100)
**High resolution CT scan**		
Nodules	27/44 (61)	15/27 (56)
Infiltrates	22/ 44 (50)	14/22 (64)
Bronchiectasis	15/44 (34)	10/15 (67)
Consolidation	13/44 (30)	8/13 (62)
Ground glass infiltrates	11/44 (25)	10/11 (91)
Emphysematous changes	8/44 (18)	7/8 (88)
Cavities	7/44 (16)	5/7 (71)
Tree in bud (Fig 3)	7/44 (16)	5/7 (71)
Pleural effusion	4/44 (9)	2/4 (50)
Hilar adenopathies	4/44 (9)	1/4 (25)
Lobar predominance on CT scan***		
Upper lobe	23/44 (52)	15/23 (65)
Lower lobe	22/44 (50)	15/22 (68)
Middle lobe	12/44 (27)	8/12 (67)

Patients meeting diagnostic criteria include those with positive *M*. *simiae* cultures who met the ATS criteria for the diagnosis of infection.

*Numbers indicate n/N (%) unless otherwise specified

*** Some patients had involvement of more than one lobe

#### Pathology

Only one patient underwent trans-bronchial biopsy. Histopathology revealed chronic inflammation with sub-pleural fibrosis; however, the biopsy from this specimen was not sent for mycobacterial culture.

#### Diagnostic criteria

The 2007 ATS guidelines for the diagnosis, treatment, and prevention of NTM diseases were adopted in this study [2]. Twenty-four of the 51 patients (47%) met the ATS criteria for NTM disease. In the remaining 27 patients, one or more of the clinical, radiographic, and microbiologic criteria were absent. These results are detailed in Tables 1 and 2.

Overall, eleven of the 24 patients who met the ATS criteria for NTM disease received treatment. An additional 6 patients who did not meet the ATS criteria were also treated. However, as will be noted later, 4 out of the 17 treated patients started therapy late during their disease course, which might have influenced their outcome.

#### Drug susceptibility testing

Drug susceptibility testing was performed on 17 of the total 51 (33%) *M*. *simiae* isolates at Mayo clinic, USA. Specimens from the rest of the patients were not sent for susceptibility testing due to financial reasons. Among patients who received early treatment, only 8 (62%) had susceptibility testing done.

The *in vitro* susceptibility results of the tested isolates are listed in Table 3. Ninety four percent of the isolates were sensitive to clarithromycin. The susceptibility to amikacin was high (88%), while only 19% and 30% of the isolates were susceptible to TMP/SMX and moxifloxacin respectively. Moreover, none were sensitive to ciprofloxacin. Two of the isolates that were resistant to all tested antibiotics except clarithromycin were tested against clofazimine and found to be susceptible.

**Table 3:** *In vitro* resistance of the 17 *M*. *simiae* isolates to the tested antibiotics (MIC mcg/ml).

Antibiotics*	MIC_50_	MIC_90_	Range	% of Resistance ^
Ciprofloxacin	8	16	8->16	100
Moxifloxacin	4	8	1->8	70
Clarithromycin	16	16	4–32	6
Amikacin	16	64	16–64	12
TMP/SMX	8/152	8/152	2->8/38-152	81
Streptomycin	64	>64	16->64	100
Linezolid	32	64	32–64	100
Ethambutol	> 16	>16	8->16	100
Isoniazid	8	>8	4–6	100
Rifampin	>8	>8	>8->8	100
Rifabutin	8	>8	4->8	100

MIC in mcg/ml of 17 isolates

* Clofazimine susceptibility was only done on 2 isolates, as it was not part of the initial routine panel and both isolates turned out to be susceptible.

**^** *M*. *simiae* isolates were considered susceptible or resistant according to MIC cut off used in Mayo clinic

#### Treatment and follow-up

The most commonly used drug regimen was clarithromycin in combination with TMP/SMX or moxifloxacin. Clarithromycin with amikacin were also used but less frequently. In addition, clarithromycin with clofazimine were used in two patients. The duration of treatment ranged from 6 to 24 months. Only one of the patients was maintained on treatment for a longer period, reaching up to 10 years.

To simplify our analysis, patients were divided into 4 cohorts depending on whether they met the ATS criteria for true NTM infection and whether they received early treatment, defined as treatment immediately after *M*. *simiae* detection (Table 4). Decision to begin treatment was based on an individual approach. For most patients, no specific guidelines were followed, and therapy was started based on physicians’ judgment of symptoms severity. Amongst the 10 patients in group A (met ATS criteria and received early treatment), 4 patients noted improvement or stabilization of symptoms. Among these, 2 patients received a combination of clofazimine and clarithromycin and showed significant improvement in their respiratory symptoms 3 months into therapy; however, both patients reported skin hyperpigmentation after being on treatment for 5 months.

**Table 4:** Outcome of patients with positive cultures for *M*. *simiae* isolates.

		Group A (n = 10)	Group B (n = 14)	Group C (n = 3)	Group D (n = 24)	Total
Improvement or stabilization		4	4	1	5	14
Persistence or recurrence	Related	1	1^b^	-	1^a^	2
	Unrelated	1^a^	-	1^c^	-	1
Death	Related	2	1	-	-	5
	Unrelated	1	1	-	2^d^	2
	Undetermined	1	2	1	1	5
Lost to follow up		-	5	-	15	20

Group A: patients who met ATS guidelines for true NTM infection and received early treatment.

Group B: patients who met ATS guidelines for true NTM infections but did not receive early treatment.

Group C: patients who did not meet ATS guidelines for true NTM infections but received early treatment.

Group D: patients who did not meet ATS guidelines for true NTM infections and did not receive early treatment.

^a^ Patient with biopsy showing sarcoidosis

^b^ Required late treatment but was eventually lost to follow-up

^c^ Patient with biopsy showing hypersensitivity pneumonitis

^d^Two patients in this group received treatment late into their disease but ended up dying

Among the 14 patients in group B (met ATS criteria but didn’t receive early treatment), 4 remained stable for several years showing no progression of their disease, and one patient died with *M*. *simiae* meningitis before receiving treatment.

In group C (didn’t meet ATS criteria but received early treatment), 1 patient had an alternative diagnosis for persistent symptoms.

Moreover, among the 24 patients in group D (didn’t meet ATS criteria and didn’t receive early treatment), 15 patients were lost to follow-up.

Of note, some patients in groups B and D received treatment late in their disease course due to persistence of symptoms. Further details on follow-up are shown in Table 4.

## Discussion

*M*. *simiae* is a frequent colonizer of the lung, and is not always considered pathogenic especially in immunocompetent patients [13]. This is largely supported by the two pseudo-outbreaks reported in the literature related to water exposure; the first was in San Antonio where *M*. *simiae* was recovered from the water supply of a hospital building and a patient's home [10]; and the second was due to contamination of the water reservoir of a hospital building in Houston [10].

*M*. *simiae* was isolated from the respiratory specimens in almost all patients. Based on the 2007 ATS guidelines, 47.1% (24/51) of the patients in this study had true *M*. *simiae* infection, which is a significant number compared to the reported rates of 21% by Van Ingen et al. [4] and 9–24% by Hashemi-Shahraki et al. [3].

*M*. *simiae* infection is mostly seen in the elderly patient population with no clear data regarding sex distribution [4, 14]. Earlier studies showed that NTM disease mostly occurs in patients with diabetes [15], cardiovascular disease, or malignancy [2]. In this series, elderly male patients were more prone to develop the infection. Furthermore, most of the patients had no clear occupational exposure. Comorbid conditions were often reported, especially chronic lung disease, diabetes, and malignancy. Latent tuberculosis (TB) was diagnosed in two of the patients of this series and active TB in one, but there was no documentation concerning the drug regimen received by these patients. Furthermore, tuberculin skin test (TST) was rarely performed since TB was not suspected. Previously, TST was shown to be positive in 76.9% of patients with *M*. *simiae* infections in a TB referral center in one study [15], and to reach an average value of 20 mm in children in another study [10].

In previous reports, *M*. *simiae* infrequently caused clinical disease [2]. It rarely affected immunocompetent patients and was mainly described in patients with acquired immunodeficiency syndrome (AIDS) or those with underlying pulmonary diseases like TB [15], bronchiectasis and COPD [1], silicosis [13], cystic fibrosis, and pneumoconiosis [16]. Likewise, most patients of this series had underlying COPD, or asthma. *M*. *simiae* was recovered from respiratory specimens in almost all of the studied subjects showing that infections mainly affect the respiratory system, which is consistent with previous publications [16].

Symptoms frequently reported in NTM infections are non-specific and can be seen in TB or fungal pulmonary infections. They include sweating, weight loss, low-grade fever, productive cough, and hemoptysis [15]. In this series, cough and sputum production were the most commonly encountered symptoms. Night sweats, hemoptysis, and weight loss were less frequently seen.

Very few cases of extra-pulmonary *M*. *simiae* infections have been reported in the literature [17]. These include parotid gland infection [18], skin lesions [10], genitourinary tract infection [7, 19], disseminated disease [2], localized lymphadenitis [10], and vertebral osteomyelitis [18, 19]. Furthermore, although extra-pulmonary disease is particularly rare in immunocompetent patients [4], a disseminated infection was detected in one of the immunocompetent patients in this study who presented with fatigue followed by fever, confusion, and interstitial lung findings. *M*. *simiae* was identified in both CSF and respiratory specimens and the patient was ultimately diagnosed with *M*. *simiae* respiratory infection with a concomitant CNS invasion [6].

Previous cases of disseminated *M*. *simiae* infections in HIV patients were reported [6, 20]. Valero et al. described a case of disseminated infection in an HIV patient with CNS lymphoma where the organism was isolated from brain tissue [5]. In this study, only one patient was known to be HIV positive; however, his infection was restricted to the lungs.

Existing data on common radiographic findings are mixed. Baghaei et al. showed that nodular lesions were found in 100% of patients, and bronchiectasis and cavities in 84% and 88%, respectively [15]. Furthermore, Baghizadeh et al. showed that the most common findings were nodular lesions (100%), and bronchiectasis (85.29%), in addition to other findings such as para-tracheal and hilar lymphadenopathy (44%), pleural effusion (20.6%), and pleural thickening (58.8%) [21]. In the latter study, the right middle lobe was the most commonly involved (50%), followed by the lingula (47%), and the right upper lobe (41%) [21]. On the other hand, Shitrit et al. noted cavitary disease in 3%, pulmonary infiltrates in 57%, and pleural effusion in 16%, along with involvement of the middle and lower lobes in 55% [1]. For this series of 51 patients, data were not available in all due to the retrospective nature of the study. Infiltrates were the most common findings on CXR, while nodules, infiltrates and bronchiectasis were mostly seen on CT scan. Notably, no predilection for upper versus lower lobes was seen (Table 2), hence, no conclusions can be drawn regarding the typical *M*. *simiae* respiratory infection radiological findings.

The most common histopathology associated with *M*. *simiae* pulmonary disease consists of the classical tuberculous-like granulomas with varying degrees of necrosis [17]. The presence of non-necrotic granulomas can also be expected in NTM infections [17]. Lung biopsy was performed in only one patient which showed chronic inflammation with sub-pleural fibrosis and reactive pneumocyte hyperplasia.

Treatment of *M*. *simiae* infection is very challenging since little information is known about the best regimen and duration [10, 17]. In addition, the *in vitro* susceptibility results do not necessarily correlate with *in vivo* activity [22, 23]; thus, physicians should weigh the risk of adverse effects against the benefit of treatment before deciding on initiating therapy [20].

In this study, drug susceptibility testing guided the treatment regimen in 8 of the 13 patients who received early treatment. The most common drug regimens included clarithromycin in different combinations with TMP/SMX, moxifloxacin, and amikacin. The duration of treatment in this study ranged from 6 to 24 months. A follow-up culture was not performed in all patients; this is not in accordance with the ATS recommendations [2], which state clearly that treatment of pulmonary disease should be continued for one year following the first negative respiratory culture. Moreover, Cruz et al. and Hankins et al. suggested a duration ranging between 6 months and 1 year for extra-pulmonary disease [10, 14].

Drug susceptibility testing should be requested for every *M*. *simiae* isolate [22] however, this was not performed in all isolates of this study due to financial reasons. *M*. *simiae* isolates from this study were mostly susceptible to amikacin and clarithromycin and less susceptible to moxifloxacin and ciprofloxacin, unlike the results by Van Ingen et al., which showed susceptibility rates of 14%-40% to amikacin, 91% to clarithromycin, 64%-87% to moxifloxacin, and 33%-62% to ciprofloxacin [22]. These findings highlight the variable susceptibly profile of this pathogen in different geographic locations and emphasizes on the need to perform susceptibility testing before initiating therapy. There remains, however, little data that correlates *in vitro* susceptibility with response to therapy.

In this study, a triple drug regimen, including clarithromycin and amikacin in association with TMP/SMX or moxifloxacin as per the susceptibility results, was preferred. According to the ATS guidelines, a regimen similar to that used for *M*. *avium* complex is advised [2]. Another regimen including clarithromycin combined with moxifloxacin and another susceptible drug (e.g. clofazimine, TMP/SMX, amikacin, streptomycin) has also been recommended [2, 22]. Furthermore, the effect of adding clofazimine to amikacin was previously explored and showed a synergistic activity against *M*. *simiae* isolates, even if they had limited sensitivity to amikacin [23]. This was explained by the Trojan horse effect of clofazimine on the cell wall of mycobacteria [23]. Although there is data to suggest possible synergy of combining clofazimine with amikacin, such synergistic effect of combining clarithromycin with clofazimine has not been tested. This combination was used in two patients in this series whose *M*. *simiae* isolates were only sensitive to clarithromycin and clofazimine with a successful outcome.

Moreover, Maoz et al. suggested a four-drug regimen containing clarithromycin, ethambutol, rifabutin and streptomycin [24], which might not be applicable in Lebanon since most of the isolates in this country were resistant to the latter three drugs.

No firm conclusions about the best regimen and duration of treatment can be made in cases of extra-pulmonary infections, due to the limited number of such cases in this cohort.

Upon follow-up, 5 of the 13 patients receiving early treatment noted clinical amelioration in their symptoms. Interestingly, out of the 14 patients in group B, 4 remained stable for several years, showing no disease progression. This proves that even if patients meet the 2007 ATS criteria for *M*. *simiae* infection, they may remain stable and not require therapy in view of the chronic nature of the infection. Patients can be closely followed up and therapy initiated once there is clinical or radiological worsening. Moreover, 2 treated patients were suspected of having an alternative diagnosis; hence, other conditions should be excluded before attributing symptoms to *M*. *simiae* infection [2]. Of note, several patients were lost to follow-up, especially in the untreated groups.

It is interesting to know that isolation of *M*. *simiae* seems to be restricted to certain geographic areas as mentioned above mainly Iran, Cuba, Israel and Arizona, but the ecology behind this is not well understood [25, 26]. It is possible that some common environmental factors including temperature and humidity might be playing a role. Since this organism has been previously isolated from water sources following pseudo-outbreaks, further studies including water cultures are needed to better assess the epidemiology and ecology of *M*. *simiae* in geographic areas where infections with this pathogen are described. Moreover, future molecular studies on environmental and clinical isolates could also help us better understand the epidemiology of this infection.

## Conclusion

In Lebanon, *M*. *simiae* is being encountered with increasing frequency with at least 47% of the isolates representing a true infection. Strains of *M*. *simiae* were multidrug resistant, had different susceptibility profiles than previously published studies, and were mostly susceptible to clarithromycin and amikacin. Patients should be closely followed up to decide on the need to treat and on the proper timing to initiate therapy after recovery of *M*. *simiae* from respiratory specimens. Future epidemiologic and molecular studies will hopefully shed more light on the understanding of the epidemiology of this infection.
